# SEQUENTIAL ANALYSIS OF DEMENTIA FAMILY DYADIC COMMUNICATION: CARE RECIPIENT CHALLENGING/NEUTRAL COMMUNICATION

**DOI:** 10.1093/geroni/igae098.1765

**Published:** 2024-12-31

**Authors:** Sohyun Kim, Will Chen, Shan Sun-Mitchell

**Affiliations:** The University of Texas Arlington, Arlington, Texas, United States; The University of Texas Arlington, Arlington, Texas, United States; The University of Texas Arlington, Arlington, Texas, United States

## Abstract

Care-recipient challenging communication is nonresponsive, resistive, withdrawing, and critical communication showing disengagement. Care-recipient neutral communication is nonverbal behavior either engaging or challenging depending on the context. This study examined temporal relationships between antecedent care-recipient challenging and neutral communication and subsequent family caregiver communication (facilitative, disabling, and neutral) during 75 in-home care video observations. Secondary analysis of 5- to 60-second timed-window analysis with 5-second intervals was conducted. 95% confidence intervals, p-values, and Yule’s Q statistics were used. The results showed that all types of family caregiver communication were less likely to occur at differing frequencies within each time window following care-recipient challenging verbal and neutral communication. Caregiver facilitative verbal, disabling nonverbal, and neutral communication were less likely to occur within all time widows after care-recipient challenging verbal communication (OR = 0.32 – 0.86, p < 0.001 – p = 0.001-0.037, Yule’s Q = -0.106 – -0.519). Caregiver facilitative and disabling communication were less likely to occur until 35 seconds after care-recipient neutral communication (OR = 0.34 – 0.87, p < 0.001 – p = 0.002 – 0.044, Yule’s Q = 0.093 – 0.459). Care-recipient challenging verbal and neutral communication were significantly associated with a decreased likelihood of subsequent caregiver communication, with a small to large probability of non-occurrence. These results highlight that caregivers often fail to respond to care-recipient challenging and neutral communication. Healthcare providers and researchers should prioritize providing caregiver education focusing on awareness and support of challenging verbal behaviors and other nonverbal behaviors of persons living with dementia.

